# The HA4M dataset: Multi-Modal Monitoring of an assembly task for Human Action recognition in Manufacturing

**DOI:** 10.1038/s41597-022-01843-z

**Published:** 2022-12-02

**Authors:** Grazia Cicirelli, Roberto Marani, Laura Romeo, Manuel García Domínguez, Jónathan Heras, Anna G. Perri, Tiziana D’Orazio

**Affiliations:** 1 Institute of Intelligent Industrial Technologies and Systems for Advanced Manufacturing, National Research Council of Italy, Bari, Italy; 2 Department of Mathematics and Computer Science, Universidad de La Rioja, Logroño, Spain; 3 Department of Electric and Information Engineering, Polytechnical University of Bari, Bari, Italy

**Keywords:** Industry, Engineering

## Abstract

This paper introduces the Human Action Multi-Modal Monitoring in Manufacturing (HA4M) dataset, a collection of multi-modal data relative to actions performed by different subjects building an Epicyclic Gear Train (EGT). In particular, 41 subjects executed several trials of the assembly task, which consists of 12 actions. Data were collected in a laboratory scenario using a Microsoft® Azure Kinect which integrates a depth camera, an RGB camera, and InfraRed (IR) emitters. To the best of authors’ knowledge, the HA4M dataset is the first multi-modal dataset about an assembly task containing six types of data: RGB images, Depth maps, IR images, RGB-to-Depth-Aligned images, Point Clouds and Skeleton data. These data represent a good foundation to develop and test advanced action recognition systems in several fields, including Computer Vision and Machine Learning, and application domains such as smart manufacturing and human-robot collaboration.

## Abstract (summary)

**Table:** 

Measurement(s)	human actions in manufacturing context
Technology Type(s)	Microsoft Azure Kinect Camera

## Background & Summary

Human action recognition is an active topic of research in computer vision^1,2^ and machine learning^3,4^ and vast research work has been carried out in the last decade, as seen in the existing literature^5^. Moreover, the recent wide-spread of low-cost video camera systems, including depth-cameras^6^, has strengthened the development of observation systems in a variety of application domains such as video-surveillance, safety and smart home security, ambient assisted living, health-care and so on. However, little work has been done in human action recognition for manufacturing assembly^7–9^ and the poor availability of public datasets limits the study, development, and comparison of new methods. This is mainly due to challenging issues such as between-action similarity, the complexity of actions, the manipulation of tools and parts, the presence of fine motions and intricate operations.

**Fig. 1:**
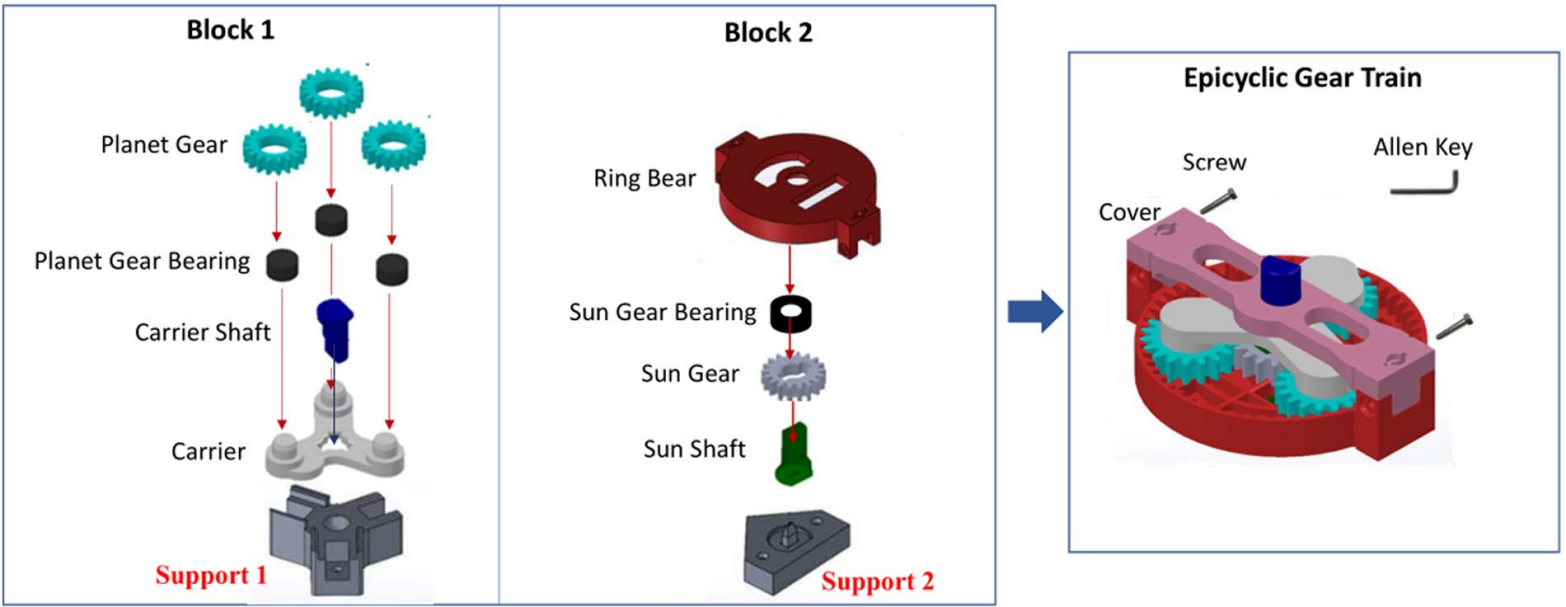
Components involved in the assembly of the Epicyclic Gear Train. The CAD model of the components is publicly available at^44^.

The recognition of human actions in the context of intelligent manufacturing is of great importance for various purposes: to improve operational efficiency^8^; to promote human-robot cooperation^10^; to assist operators^11^; to support employee training^9,12^; to increase productivity and safety^13^; or to promote workers’ good mental health^14^. In this paper, we present the Human Action Multi-Modal Monitoring in Manufacturing (HA4M) dataset which is a multi-modal dataset acquired by an RGB-D camera during the assembly of an Epicyclic Gear Train (EGT) (see Fig. 1).

The HA4M dataset provides a good base for developing, validating and testing techniques and methodologies to recognize assembly actions. Literature is rich in RGB-D datasets for human action recognition^15–17^ prevalently acquired in indoor/outdoor unconstrained settings. They are mostly related to daily actions (such as walking, jumping, waving, bending, etc.), medical conditions (such as headache, back pain, staggering, etc.), two-person interactions (such as hugging, taking a photo, finger-pointing, giving object, etc.), or gaming actions (such as forward punching, tennis serving, golf swinging, etc.). Table 1 reports some of the most famous and commonly used RGB-D datasets on human action recognition describing their principal peculiarities.

**Table 1:** Some popular publicly available RGB-D Datasets for 3D Action Recognition.

Dataset	Sensors	Environment	Data Modalities	Actions
NTU RGB + D 120^30,31^	Microsoft Kinect v2	Cluttered Indoor	RGB Videos, Depth Sequences, 3D Skeleton Joints, IR Frames	Daily, Medical, Two People Interaction
SYSU 3DHOI^32^	Microsoft Kinect v1	Cluttered Indoor	RGB Videos, Depth Sequences, 3D Skeleton Joints	Daily
Drive&Act^33^	Five NIR cameras and One Microsoft Kinect	Static Driving Simulator	RGB, IR and Depth Data	Driver Behaviors
UE-HRI^34^	Two RGB cameras and one 3D sensor	Cluttered Indoor	RGB and Depth Frames	Human Robot Interaction
MoCa^35^	Three RGB cameras and Vicon Motion Capture System	Laboratory	RGB, 3D Skeleton Joints	Cooking
Grasping Dataset^36^	GoPro Hero 4 Camera, SoftKinetic Camera and IMU sensors	Living Room and Kitchen	RGB, Dept and IMU Data	Cooking, Housework
MSR-Action3D^37^	Microsoft Kinect v1	Cluttered Indoor	Depth Sequences, 3D Skeleton Joints	Daily
MSR Daily ACtivity 3D^38^	Microsoft Kinect v1	Cluttered Indoor	RGB Videos, Depth Sequences, 3D Skeleton Joints	Daily
UT-Kinect^39^	Microsoft Kinect v1	Cluttered Indoor	RGB Videos, Depth Sequences, 3D Skeleton Joints	Daily
RGBD-HuDaAct^40^	Microsoft Kinect v1	Laboratory	RGB Videos, Depth Sequences	Daily

They prevalently collect RGB, Depth and 3D skeleton joints information relative to actions from daily activities conducted in indoor environments such as office-like, laboratory environments, or living rooms.

To the best of the authors’ knowledge, few vision-based datasets exist in the context of object assembly. Researchers usually build their own datasets on private video data^7,18^. Table 2 compares the proposed HA4M dataset with existing datasets on assembly action recognition. As shown in Table 2, the proposed HA4M features various main contributions:

- **Data Variety:** The HA4M dataset provides a considerable variety of multi-modal data compared to existing datasets. Six types of simultaneous data are supplied: RGB frames, Depth maps, IR frames, RGB-to-Depth-Aligned frames, Point Clouds and Skeleton data. These data allow the scientific community to make consistent comparisons among processing approaches or machine learning approaches by using one or more data modalities.
- **Action Variety:** The HA4M dataset presents a wide variety in the action execution considering the high number of subjects (41) performing the task, the high number of actions (12), the different order followed by the subjects to perform the actions, and the interchangeably use of both hands.
- **Fine-grained Actions:** Actions present a high granularity as there is a subtle distinction between parts to be assembled and between actions that appear visually similar.
- **Challenging Issues:** The components to be assembled and the actions are very similar and symmetrical. Then the action recognition task requires a high level of context understanding and a significant object-tracking ability. The environmental scenario of the dataset is realistic and does not change over time, as usually happens in industrial assembly contexts. Therefore, recognizing different actions is very challenging as it depends only on tracking the movements of the arms of the operator. In addition, the dataset comprises untrimmed videos containing actions performed consecutively in different orders. Temporal action segmentation is crucial in high-level video understanding. So, the proposed dataset can be used to test action segmentation as well as action recognition tasks.

**Table 2:** Comparison between the proposed HA4M dataset and existing vision-based datasets on assembly actions.

Dataset	Visual Sensors	Environment	Data Modalities	Task
Assembly101^41^	Eight RGB Cameras mounted on a scaffold around a table and four monochrome cameras mounted on an headset	Laboratory	RGB frames, 3D hand poses	Assembly and Disassembly of toy vehicles
Meccano^42^	One Intel RealSense SR300 camera mounted on an headset	Laboratory	RGB videos	Assembly of a toy motorbike
IKEA-ASM^43^	Three Microsoft Kinect v2	Offices, Labs and Family Homes	RGB videos, Depth videos, 3D Skeleton Joints	Furniture Assembly
HA4M	Microsoft Azure Kinect	Laboratory	RGB frames, Depth maps, IR frames, RGB-Depth-Aligned frames, Point Clouds, Skeleton Data	Assembly of an EGT

For each dataset, information about the cameras used for data acquisition, the type of environment where acquisitions were made, the type of provided data and the assembly task are given.

## Methods

### Study design

In the proposed dataset, a Microsoft Azure Kinect^19,20^ camera acquires videos during the execution of the assembly task. The Azure Kinect camera offers improved accuracy than other affordably RGB-D sensors implementing Time of Flight (ToF) principles^21^, making the Azure Kinect one of the best solution for indoor human body tracking in manufacturing scenarios^22–24^.

The assembly of an EGT involves three phases (Fig. 1): first, the assembly of Block 1 and Block 2 separately and then the final building of both blocks. The EGT is made up of a total of 13 components: eight components to build Block 1, four components to build Block 2, and a cover to assemble Block 1 and Block 2. Finally, two screws fix the two blocks with an Allen key, thus obtaining the EGT. In Fig. 1, the two supports used to facilitate the assembly of each block are also shown. Table 3 lists the individual components and the actions necessary for assembling Block 1, Block 2 and the whole EGT, respectively. The total number of actions is 12, divided as follows: four actions for building Block 1; four actions for building Block 2; and four actions for assembling the two blocks and completing the EGT. As can be seen in Table 3, some actions are performed more times as there are more components of the same type to be assembled: actions 2 and 3 are executed three times, while action 11 is repeated two times. Finally, a “don’t care” action (ID = 0) has been added to include transitions or unexpected events such as the loss of a component during the assembly.

**Table 3:** List of components and actions needed to build Block 1, Block 2 and EGT, respectively.

Components			Actions	
	Quantity	Description	Action ID	Action Description
Block 1	3	Planet Gear	1	Pick up/Place Carrier
	3	Planet Gear Bearing	2	Pick up/Place Gear Bearings (×3)
	1	Carrier Shaft	3	Pick up/Place Planet Gears (×3)
	1	Carrier	4	Pick up/Place Carrier Shaft
Block 2	1	Ring Bear	5	Pick up/Place Sun Shaft
	1	Sun Gear Bearing	6	Pick up/Place Sun Gear
	1	Sun Gear	7	Pick up/Place Sun Gear Bearing
	1	Sun Shaft	8	Pick up/Place Ring Bear
EGT	1	Block 1	9	Pick up Block 2 and place it on Block 1
	1	Block 2	10	Pick up/Place Cover
	1	Cover	11	Pick up/Place Screw (×2)
	2	Screws	12	Pick up Allen Key, Turn both screws, Return Allen Key and the EGT

First, the assembly of Block 1 (action IDs 1 to 4), then Block 2 (action IDs 5 to 8) and finally the EGT (action IDs 9 to 12).

### Acquisition setup

**Fig. 2:**
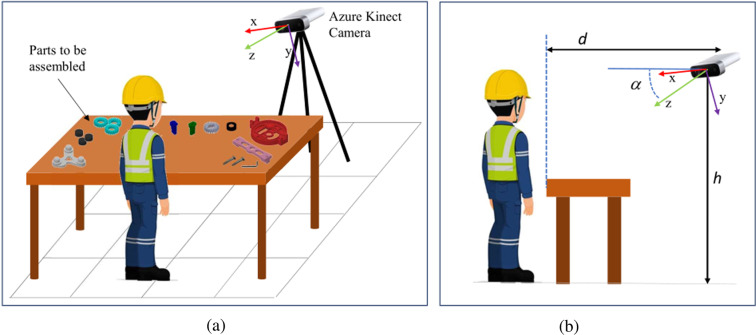
Sketch of the acquisition setup: (**a**) a Microsoft® Azure Kinect is placed in front of the operator and the table where the components are spread over; (**b**) setup specifications.

The experiments took place in two laboratories (one in Italy and one in Spain). The acquisition setup is pictured in Fig. 2. A Microsoft Azure Kinect® was placed on a tripod in front of the operator at a height *h* = 1.54 *m* above the floor and a horizontal distance *d* = 1.78 *m* from the far border of the table. The camera is tilted down to an angle *α* = 17 (see Fig. 2b). As shown in Fig. 2a, the individual components to be assembled are spread on a table in front of the operator and are placed according to the order of assembly. The operator can pick up one component at a time to perform the assembly task standing in front of the table.

**Fig. 3:**
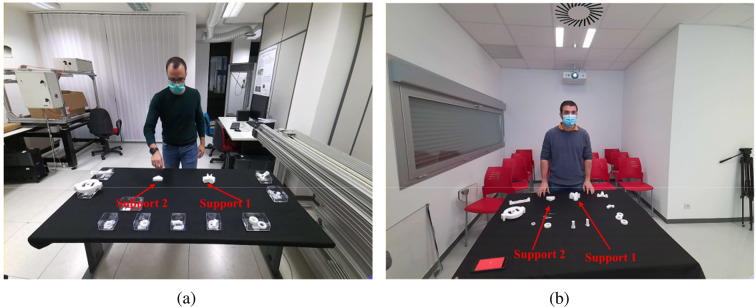
Typical video frames acquired by the RGB-D camera in the (**a**) “Vision and Imaging Laboratory” of STIIMA-CNR in Bari (Italy) and at the (**b**) “Department of Mathematics and Computer Science”, Universidad de La Rioja, Logroño (Spain).

Two typical RGB frames captured by the camera in each laboratory are shown in Fig. 3. The working table is covered by a uniform tablecloth, while the components are arranged into boxes or spread over the table. In Fig. 3, the two supports, fixed on the table to facilitate the assembly of Block 1 and Block 2, are identified by arrows. Block components can be white over a black tablecloth or black over a white tablecloth. In both cases, the items are well visible over the table.

### Study participants

The HA4M dataset contains 217 videos of the assembly task performed by 41 subjects (15 females and 26 males). Their ages ranged from 23 to 60 years. All the subjects participated voluntarily and were provided with a written description of the experiment. Additionally, they read and signed an informed consent form, conserved at the “Institute of Intelligent Industrial Systems and Technologies for Advanced Manufacturing” (STIIMA), of the “National Research Council” (CNR) of Italy. The study and experiments were approved by the institutional Ethics Committee of CNR with Notification n. 0013464-2022. The subjects were first instructed about the sequence of actions to perform to build the EGT. However, where possible, differences in assembly order were allowed. As an example, actions 2 and 3 can be performed three times in sequence (i.e. 2, 2, 2, 3, 3, 3) or alternatively (i.e. 2, 3, 2, 3, 2, 3). Furthermore, each subject was asked to execute the task several times and to perform the actions as preferred (e.g. with both hands), independently of their dominant hand.

### Data annotation

Data annotation concerns the labeling of the different actions in video sequences. The annotation of the actions has been manually done by observing the RGB videos frame by frame, and cross-checked by two researchers having different backgrounds, engineering or computer science. The start frame of each action is identified as the subject begins to move the arm to the component to be grasped. The end frame, instead, is recorded when the subject releases the component, so that the next frame becomes the start of the subsequent action. The total number of actions annotated in this study is 4124, considering that actions 2 and 3 are performed three times in each video, whereas action 11 is performed 2 times (see Table 3). Furthermore, the “don’t care” action has been annotated 435 times in all the videos.

**Fig. 4:**
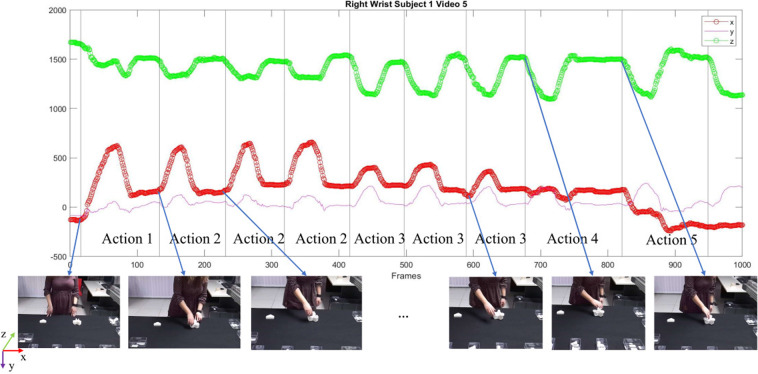
Check of annotation procedure. The plot reports the trajectories of the (*x*, *y*, *z*) coordinates of the right wrist of a right-handed subject in the first 1000 frames of an acquired video. The vertical lines identify the start frame of the actions annotated manually. Some relative RGB frames are also displayed. Frames have been cropped for visualization purposes.

Once the manual annotation was completed, the wrist joints of both hands were analyzed to further check the manual labeling. Referring to Fig. 4, which shows the movement of the right wrist during the first 1000 frames of a sample video, local points of curvature variation of the *x* and *z* coordinates of the wrist joints can be considered as the points of action change. These points coincide with the start frame of each action (vertical lines in Fig. 4) obtained by manual video annotation. It is worth noticing that the *y* coordinate does not give information for annotation check since it represent the joint height, typically constant and close to the table height during all actions.

## Data Records

**Fig. 5:**
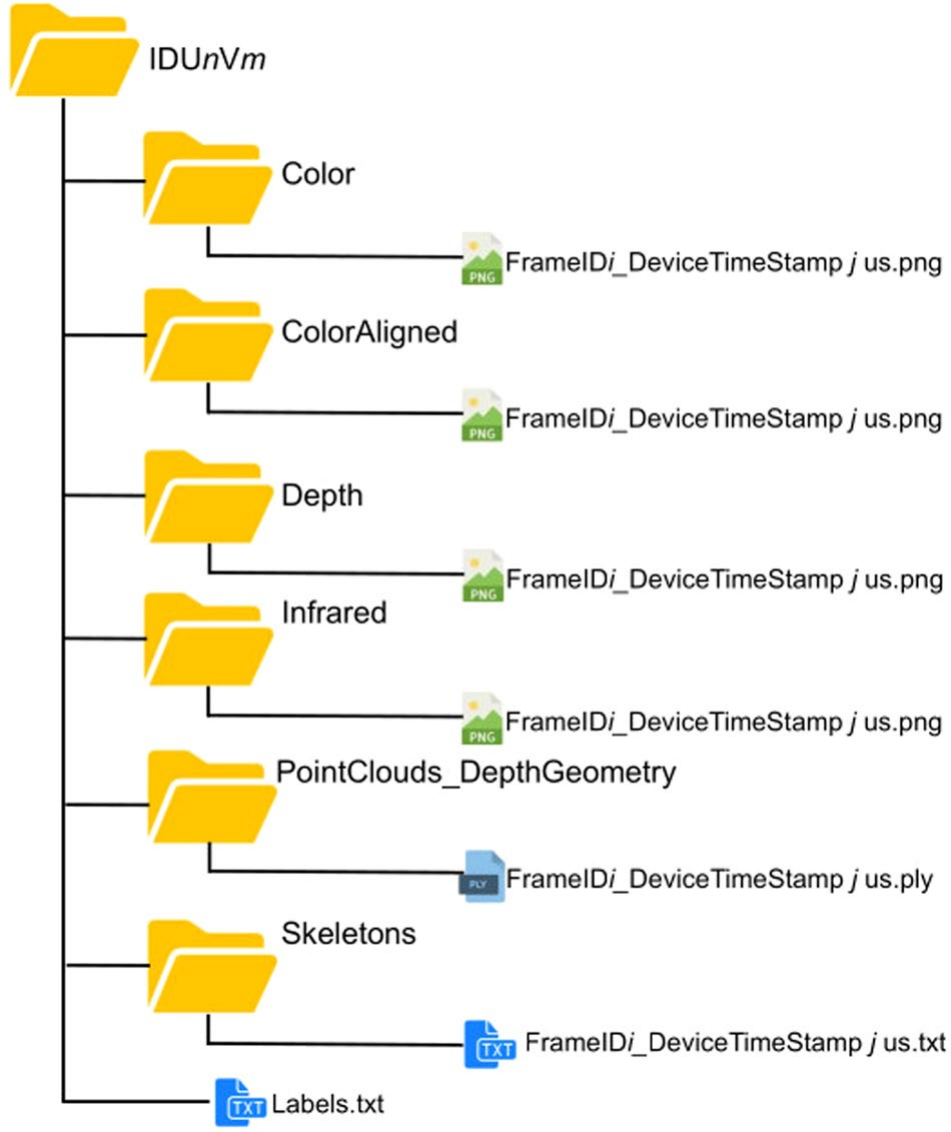
Dataset structure for each subject and each video. The name of the folder “*IDUnVm*” contains the ID subject identification number *n* and the video identification number *m*. This folder contains the annotation file (“Labels.txt”) and 6 sub-folders containing the RGB frames, the RGB-to-Depth-Aligned (RGB-A) frames, the Depth frames, the IR frames, the Point Clouds and the Skeleton data, respectively.

The dataset is publicly available at “https://baltig.cnr.it/ISP/ha4m” and Science Data Bank repository^25^. The size of the entire dataset is about 4.1 TB. and is organized as shown in Fig. 5. The data relative to each subject and each video are stored in a folder named *“IDUnVm”*, where *n* and *m* indices refer to the subject identification number (*n* = 1,…,41) and the video identification number, respectively. This folder contains the annotation file (“Labels.txt”) and 6 sub-folders named respectively: “Color”, “Color_Aligned”, “Depth”, “Infrared”, “Point_Clouds_DepthGeometry” and “Skeletons”. The sub-folders contain the RGB frames, the RGB-depth Aligned (RGB-A) frames, the Depth frames, the IR frames, the Point Clouds and the Skeleton data, respectively. Before accessing to the data, there is a second level of subfolders, named with the serial number of the Azure Kinect camera. For the sake of clarity, this level will be neglected in the next lines since each video is acquired by a single camera and, thus, the knowledge of its serial number will not add information to the dataset description.

The name of the files contained in each sub-folder is “FrameIDiDeviceTimeStampjus”, where *i* and *j* refer to the frame number and the timestamp, respectively, whereas “us” is the time unit (microseconds). Note that the timestamp is estimated relatively to the specific acquisition device. In the case of “Color” and “Color_Aligned” sub-folders, the timestamp is relative to the RGB sensor of the Azure Kinect. On the contrary, in the case of “Depth”, “Infrared”, “Point_Clouds_DepthGeometry” and “Skeletons” sub-folders, the timestamp in the filenames is relative to the IR sensor. The slight delay between RGB and Depth cameras is negligible, as it is on average much lower than the inverse of the frame rate of the camera.

Table 4 gives some details about the data, such as type, dimension, and file format. All the image files (RGB, RGB-A, Depth, IR) are in the PNG file format. RGB frames have 2048 × 1536 resolution and three channels of 8 bits each. Depth frames are grayscale images with 640 × 576 resolution and 16-bit channel depth. Each pixel value other than 0 represents the depth distance expressed in *mm*. IR frames have the same characteristics of Depth frames, where each pixel value other than 0 here represents the detected IR value. The RGB-A frames are RGB frames projected onto the IR sensor by internal geometrical transformation. Resulting frames have thus 640 × 576 resolution, equal to the one of the IR and Depth images. In contrast, images are stored with four channels: three 8-bit channels for the RGB values and an additional 8-bit *α* channel. *α* values can be equal to 255 or 0 depending on whether the depth information is available. Finally, the Point Cloud files are stored in the PLY file format. These are binary little-endian files that can have at most 640 × 576 = 368640 points, depending on the presence of the depth information. The files are in system of reference of the IR sensor. The 3D coordinates of the vertices are in meters, and the RGB color information is in three 8-bit uchar entries.

**Table 4:** Data information.

Data Type	Dimension	Details	File Format
RGB	2048 × 1536	3 channels (8-bit)	PNG
RGB-A	640 × 576	4 channels (8-bit)	PNG
Depth	640 × 576	1 channel (16-bit)	PNG
IR	640 × 576	1 channel (16-bit)	PNG
Point Cloud	max 368640 points	binary-little-endian files	PLY
Skeleton	32 joints	—	TXT

**Fig. 6:**
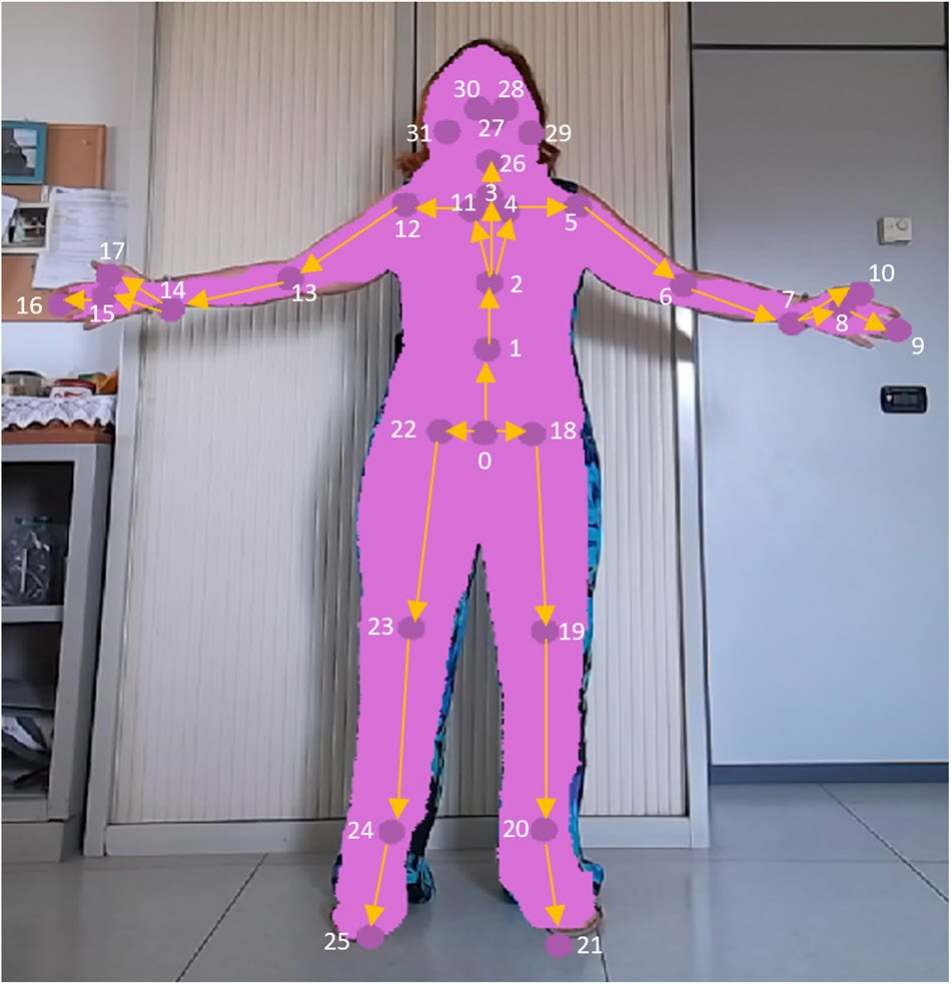
Joint locations and connections relative to the human skeleton extracted using the Microsoft Azure Kinect Body Tracking SDK v1.1.2^19^. The skeleton includes 32 joints, numbered from 0 to 31, with the joint hierarchy flowing from the center of the body to the extremities.
- **Joint Confidence Level:** the confidence level can have values equal to 0 if the joint is out of the depth range or field-of-view; 0.33 if the joint is occluded but its position is predicted; 0.67 if the joints are visible and correctly identified. This last value is the maximum confidence level in joint pose returned by the Azure Kinect Body Tracking SDK (version 1.1.2).
- **Joint 3D position:** (*X*, *Y*, *Z*) position of the joint in millimeter units. The joint position and orientation are estimated in the system of reference of the IR sensor of the Azure Kinect camera.
- **Joint 3D orientation:** the orientation, (*Qw*, *Qx*, *Qy*, *Qz*), is expressed as a normalized quaternion.
- **Joint 2D color-space and depth-space:** both Depth and RGB cameras are associated with an independent 2D coordinate system. So, each joint has 2D position coordinates in both color (*x*2*DColor, y*2*DColor*) and depth (*x*2*DDepth, y*2*DDepth*) images, respectively. If the joint is out of color or depth image, the relative coordinates assume a value of 0.

The files containing the skeleton data at each frame are in TXT format. These files exist only if a human is detected on the scene. The files contain 14 columns with the following elements:

- **Skeleton ID:** Azure Kinect Body Tracking SDK can track multiple human bodies in the scene. In our case, there is only one person in the scene, so Skeleton ID is usually 1.
- **Joint ID:** the skeleton model includes 32 joints in the range of 0–31. The joint hierarchy flows from the center of the body to the extremities, as illustrated in Fig. 6.

**Fig. 7:**
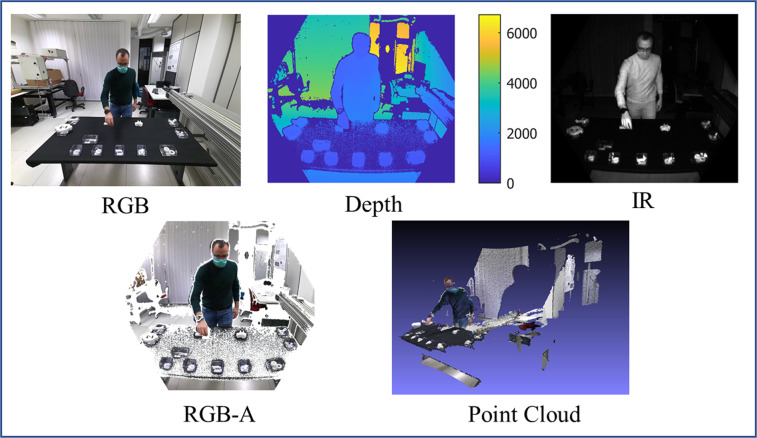
Sample frames: RGB, depth, IR, RGB-A, and point cloud. Images have been manipulated for visualization purposes.

Figure 7 shows a sample frame for each type of images: RGB, Depth, IR and RGB-A. For completeness, a representation of the relative Point Cloud is also pictured.

Each video folder has the “*Labels.txt*” file, which contains the corresponding manual annotation. This file is made of three columns: the first contains the frame number; the second contains the action ID number (in the range 0–12); the third has an integer index which refers to the repetition of the current action. This index can be 0, 1, or 2, indicating that the current action execution is the first, second, or third, respectively. Repetition numbers other than 0 are allowed in case of actions 2, 3 and 11 (see Table 3).

## Technical Validation

This section provides a statistical evaluation of the acquired data and an insight into some scientific issues that can be explored by using the HA4M dataset.

### Data assessment

This paragraph presents a spatio-temporal analysis of the actions. As a first characterization of the data, the variance of action duration is first assessed. Then, a spatial analysis of the 3D position of the wrist joints is also explored to further characterize the data. Notice that the “don’t care” action is not considered in this evaluation study as it does not contribute to the assembly of the EGT.

#### Temporal analysis

**Fig. 8:**
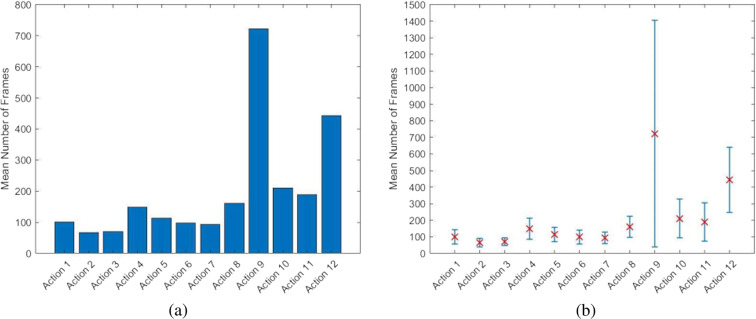
(**a**) Mean number of frames and (**b**) relative standard deviation for each action, estimated on the entire dataset.

Videos were recorded by the Azure Kinect camera at 30 frames per second (fps). Figure 8a,b show the mean number of frames with the relative standard deviation for each action over all the recorded videos. For completeness, Tables 5,6 numerically list time statistics for each action and the videos, respectively, in terms of the number of frames and execution time.

**Table 5:** Some statistics about the actions: Action Identification Number (*Column 1*); Number of the manual annotated instances (*Column 2*); Minimum Length (*Column 3*), Maximum Length(*Column 4*), Mean Length (*Column 5*) and Variance (*Column 6*) of each action in terms of number of frames; Mean Length (*Column 7*) and Variance (*Column 8*) of each action in seconds.

Action ID	Action Instances	Min Length *(frames)*	Max Length *(frames)*	Mean Length *(frames)*	Variance *(frames)*	Mean Length *(sec)*	Variance *(sec)*
1	217	8	263	100.23	42.99	3.34	1.43
2	651	22	207	66.29	26.01	2.20	0.86
3	651	25	210	70.27	23.71	2.34	0.79
4	217	63	632	148.57	62.92	4.95	2.09
5	217	48	264	113.88	42.52	3.79	1.41
6	217	37	384	98.47	42.32	3.28	1.41
7	217	38	254	93.67	35.10	3.11	1.16
8	217	54	415	161.23	63.05	5.38	2.09
9	217	114	4984	722.35	682.27	23.66	22.01
10	217	40	843	210.35	116.40	7.01	3.87
11	434	50	918	188.48	115.71	6.28	3.85
12	217	134	1488	443.70	197.60	14.7	6.58

**Table 6:** Some statistics about the videos: Minimum Length (*Column 1*), Maximum Length(*Column 2*), Mean Length (*Column 3*) and Variance (*Column 4*) of videos in terms of number of frames; Mean Length (*Column 5*) and Variance (*Column 6*) of videos in seconds.

Min Length *(frames)*	Max Length *(frames)*	Mean Length *(frames)*	Variance *(frames)*	Mean Length *(sec)*	Variance *(sec)*
997	7262	2947.31	1067.12	98.40	36.21

**Fig. 9:**
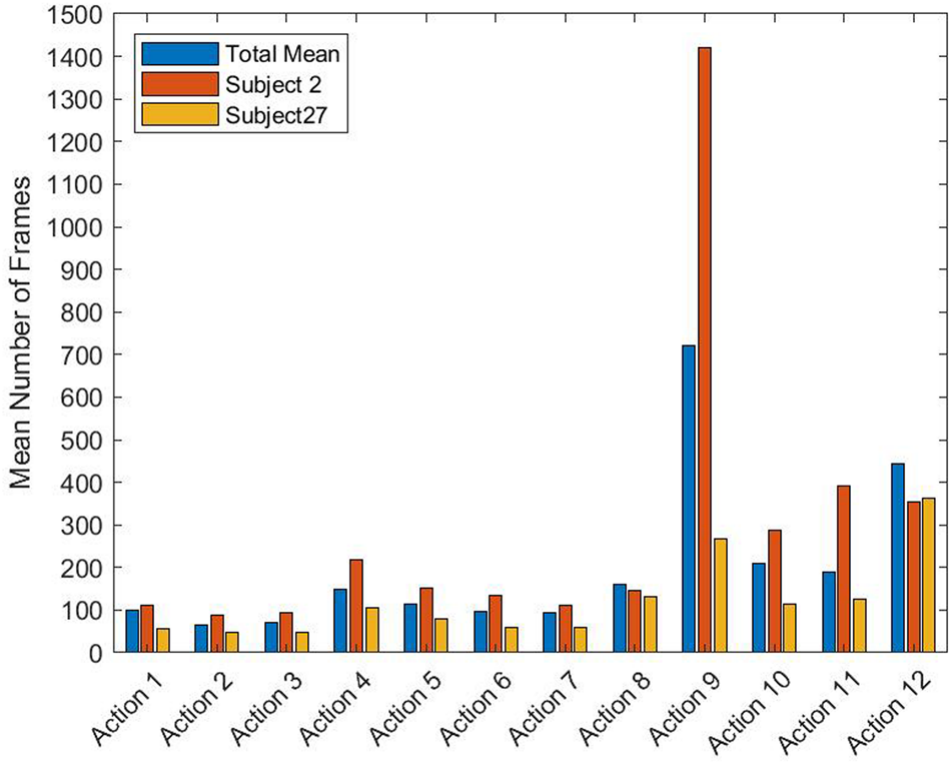
Comparative analysis of the performance of two subjects. Histograms show the mean number of frames for each action executed by subject 2 and subject 27 compared with the mean number of frames evaluated over the total dataset.

As can be seen, actions that require more time have a high variance in execution times. These actions can be more complex such as action 9 (assembly of Block 1 and 2), or can involve a longer activity such as action 12 (screw tightening). Furthermore, the subjects perform the task at their comfortable self-selected speed, so high time variance can be noticed among the different subjects. Figure 9 compares the mean number of frames for each action evaluated in the videos of two different subjects (number 2 and number 27) and the total dataset. As can be noticed, subject 2 executes the actions at a lower speed than subject 27, which, on the contrary, is very fast in task execution, even with respect to the total mean. This is mainly due to the different abilities of subjects in assembling the EGT or by accidental events, such as the loss and recovery of a component.

#### Spatial analysis

**Fig. 10:**
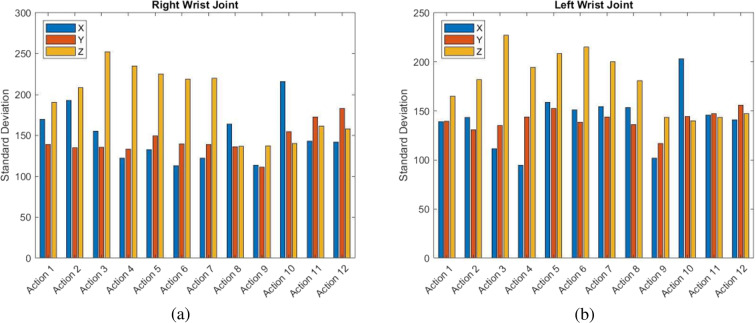
Standard deviation of the coordinates (*X*, *Y*, *Z*) of (**a**) right wrist joint and (**b**) left wrist joint of all subjects and for each action.

The analysis of the spatial movement of both wrists of all subjects is useful for getting information about the main direction and spatial displacement of each action. Figure 10a,b show the standard deviation of the coordinates (*X*, *Y*, *Z*) of the right wrist joint and the left wrist joint of all subjects and for each action, respectively. As can be noticed, different categories of actions can be identified according to the spatial properties: for instance, actions from 1 to 7 mainly evolve along the *Z* direction, whereas action 8 and 10 along the *X* direction. Finally, actions 9, 11 and 12 present comparable movements along the three directions as these actions require more spatial manipulations of the EGT. It is worth noticing that this spatial analysis can be biased by the way the subjects performed the tasks, since no precise rules were imposed to have the highest variability of the dataset. Accordingly, some subjects used their dominant hand while others used both hands interchangeably.

### Scientific issues

This section discusses some issues that can be explored using the proposed HA4M dataset in several application contexts.

#### Human centered approach in Industry 4.0

In the last years, the focus of smart manufacturing has been mainly on the transformation of manufacturing systems into new models with improved operational properties and new technologies. More recently, the focus has changed to a new perspective that puts workers at the center of the digital transformation, where technology must facilitate or improve human physical or cognitive abilities instead of replacing them^26^. As a consequence, the scientific community is very active in this domain by studying and developing intelligent systems to monitor workers to determine how they work, their pain points, and the challenges they face. So, observing the movements of human operators in the real scenario of an assembly task is very important to recognize their capabilities/competencies, especially in collaborative tasks with robots. Moreover, one of the main points of smart factory solutions is the inclusion of impaired people or people with different manual skills in production processes. The HA4M dataset represents a testbed for analysing the operative conditions of different subjects having varying skill levels. In the dataset, people with distinct ages and abilities execute complex actions in very different ways. One challenging task is the development of time-invariant action recognition methodologies capable of recognizing very different executions of the same actions. The spatial and temporal analysis of the actions presented in the previous section demonstrates the high variability of the execution of the actions, which is correlated not only to the speed of execution but also to the subjects’ ability in handling the EGT parts.

#### Multi-modal data analysis

For years, human action recognition literature has been dominated by vision-based approaches using monocular RGB videos, making action representations difficult in 3D space. Moreover, challenging issues that commonly appear in the scene, such as illumination variations, clutter, occlusions, background diversity, must be tackled to have robust recognitions. The development of low-cost technologies has made available further sensory modalities to overcome some of the challenges mentioned above^27^. The HA4M dataset provides several types of data such as depth, infrared, or point cloud extracted using the Azure Kinect sensor. Therefore, the dataset allows the research in multi-modal data integration to take advantage of the peculiarity of each sensor (RGB and IR) and overcome their intrinsic limitations.

#### Temporal action segmentation

Literature is rich of works on action recognition methodologies successfully applied to short videos analysis. In recent years, the focus has been on temporal segmentation of actions in long untrimmed videos^28^. In Industry 4.0 domain, where collaborative tasks are performed by humans and robots in highly varying conditions, it is imperative to recognize the exact beginning and ending of an action. The HA4M dataset contains long videos with multiple instances of actions performed in different ways and in different orders. Therefore, the analysis of these videos requires the recognition of action sequences. Here, the problem of the temporal segmentation of the action aims to capture and classify each action segment into an action category.

#### Human-object interaction

The analysis of videos of human-object interactions involves understanding human movements, recognizing and locating objects, and observing the effects of human movements on those objects^29^. Traditional approaches to object classification and understanding of actions relied on shape features and movement analysis. In the context of assembly tasks, the relationships between movements and handled objects can help with action recognition. Sequences of actions that manipulate similar objects (such as inserting the planet gear onto the planet gear bearing) can be aggregated to create a higher level of semantic actions. The presence of RGB images and point clouds in the HA4M dataset could allow the recognition of tools and parts with pattern recognition approaches and their relative manipulation to improve the target of action classification.

## Data Availability

The dataset has been acquired using the Multiple Azure Kinect GUI software, whose source code and the corresponding installer are available at “https://gitlab.com/roberto.marani/multiple-azure-kinect-gui”. This software is based on the Azure Kinect Sensor SDK v1.4.1 and Azure Kinect Body Tracking SDK v1.1.2^19^. In particular, the Azure Kinect SDK provides an API to record device data in a Matroska (.mkv) file containing video tracks, IMU samples, and device calibration. In this work, IMU samples are not considered. The Multiple Azure Kinect GUI software processes the Matroska file and returns the different types of data: RGB images, RGB-to-Depth-Aligned (RGB-A) images, Depth images, IR images, and Point Cloud. At the same time, exploiting the Azure Kinect Body Tracking SDK, skeletal data are stored in the corresponding TXT files. Along with the dataset, a Matlab demo code (.m file) is also provided to plot the skeletons onto the corresponding RGB images.
